# Spectroscopic and In Silico Studies on the Interaction of Substituted Pyrazolo[1,2-a]benzo[1,2,3,4]tetrazine-3-one Derivatives with c-Myc G4-DNA

**DOI:** 10.3390/ijms22116028

**Published:** 2021-06-02

**Authors:** Simone Mulliri, Aatto Laaksonen, Pietro Spanu, Riccardo Farris, Matteo Farci, Francesco Mingoia, Giovanni N. Roviello, Francesca Mocci

**Affiliations:** 1 Department of Chemical and Geological Sciences, University of Cagliari, I-09042 Monserrato, Italy; mulliri.simone@hotmail.com (S.M.); farrisric@outlook.com (R.F.); farcifarci@live.it (M.F.); 2 State Key Laboratory of Materials-Oriented and Chemical Engineering, Nanjing Tech University, Nanjing 210009, China; 3 Division of Physical Chemistry, Department of Materials and Environmental Chemistry, Arrhenius Laboratory, Stockholm University, 10691 Stockholm, Sweden; 4 Centre of Advanced Research in Bionanoconjugates and Biopolymers, Petru Poni Institute of Macromolecular Chemistry, 700487 Iasi, Romania; 5 Department of Engineering Sciences and Mathematics, Division of Energy Science, Luleå University of Technology, SE-97187 Luleå, Sweden; 6 Istituto di Chimica Biomolecolare, ICB-CNR-Trav. La Crucca 3, 07100 Sassari, Italy; pietro.spanu@cnr.it; 7 Istituto per lo Studio dei Materiali Nanostrutturati ISMN-CNR, Via U. La Malfa 153, I-90146 Palermo, Italy; francesco.mingoia@ismn.cnr.it; 8 Istituto di Biostrutture e Bioimmagini, IBB-CNR, Via Mezzocannone 16, I-80134 Naples, Italy

**Keywords:** circular dichroism, docking, molecular dynamics, c-myc, DNA quadruplexes, anticancer drugs, quadruplex stabilization

## Abstract

Herein we describe a combined experimental and in silico study of the interaction of a series of pyrazolo[1,2-a]benzo[1,2,3,4]tetrazin-3-one derivatives (PBTs) with parallel G-quadruplex (GQ) DNA aimed at correlating their previously reported anticancer activities and the stabilizing effects observed by us on c-myc oncogene promoter GQ structure. Circular dichroism (CD) melting experiments were performed to characterize the effect of the studied PBTs on the GQ thermal stability. CD measurements indicate that two out of the eight compounds under investigation induced a slight stabilizing effect (2–4 °C) on GQ depending on the nature and position of the substituents. Molecular docking results allowed us to verify the modes of interaction of the ligands with the GQ and estimate the binding affinities. The highest binding affinity was observed for ligands with the experimental melting temperatures (T_m_s). However, both stabilizing and destabilizing ligands showed similar scores, whilst Molecular Dynamics (MD) simulations, performed across a wide range of temperatures on the GQ in water solution, either unliganded or complexed with two model PBT ligands with the opposite effect on the T_m_s, consistently confirmed their stabilizing or destabilizing ability ascertained by CD. Clues about a relation between the reported anticancer activity of some PBTs and their ability to stabilize the GQ structure of c-myc emerged from our study. Furthermore, Molecular Dynamics simulations at high temperatures are herein proposed for the first time as a means to verify the stabilizing or destabilizing effect of ligands on the GQ, also disclosing predictive potential in GQ-targeting drug discovery.

## 1. Introduction

G-quadruplex (GQ) is a non-canonical secondary structure of DNA that can be found in specific regions of the genome including the telomeric ends of chromosomes and gene regulatory regions. GQs are considered promising drug targets, and compounds that are capable of binding and stabilizing this type of DNA folding would be of great benefit in anticancer therapy [1,2,3].

**Figure 1:**
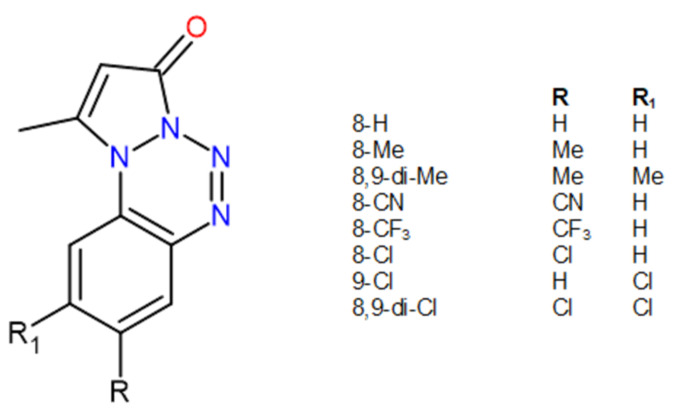
Structure of the investigated pyrazolo[1,2-a]benzo[1,2,3,4]tetrazin-3-one derivatives (PBTs).

For many classes of compounds that exhibit antiproliferative activity against tumor cell lines, the biological target is often not well known. This is the case of a class of variously substituted pyrazolo[1,2-a]benzo[1,2,3,4]tetrazin-3-one derivatives (PBTs) synthesized by some of us, which, in a preliminary National Cancer Institute (NCI) screening, showed a promising antiproliferative activity against a panel of about 60 tumor cell lines. In this screening, several PBTs, depending on the position and nature of the substituents, targeted selectively specific tumor cells or in other cases more than one in the µM or sub-µM range [4]. These results, for this tricycle scaffold, biologically unexplored so far, led us to study new candidates from this class of compounds and evaluate the biological events they are involved in, like apoptosis induction and cell cycle perturbation [5]. Once assessed for their anticancer potential, further studies became necessary in order to get insights on their mechanism of action. Thus, more specific biological targets, such as duplex DNA and topoisomerase (II) catalytic cycle, were examined, including in silico support [6]. These investigations have shown that several compounds of this class of derivatives can bind to DNA duplex, and such interactions partially explained their activity. However, these studies did not consider the possibility that the anticancer activity might be connected to the interaction and stabilizations of the DNA quadruplex structure. To explore this possibility, the interaction of eight differently functionalized PBTs (Figure 1 and Table 1) with GQ are studied in the present work, focusing on the GQ structure formed in the promoter sequence of the human c-myc oncogene [7] having the sequence: 5′-TGAGGGTGGGTAGGGTGGGTAA-3′. This GQ was shown to be associated with the promoter activity regulation and with the regulation of its transcription [8].

Aiming at deepening the knowledge on their anticancer mechanism by exploring possible relationships between the known anticancer activity of the selected PBTs compounds and their GQ binding and stabilization properties, circular dichroism (CD) spectra of c-myc GQ, either unliganded or in complex with the PBTs, were recorded at variable temperatures. CD spectroscopy is a technique typically employed to verify the formation of the GQ structure in G-rich DNAs [9,10], how the denaturing temperature is affected by the studied compounds, and thus, to quantify their effect on the GQ stability [11,12]. In order to characterize the interaction between the ligands and the GQ, docking experiments of the tested PBTs were performed against the c-myc GQ. Docking experiments allow us to verify the favored binding sites, estimate the binding affinity, and have been employed on several studies aimed at identifying GQ binders [13,14]. The effect of selected PBTs on the GQ stabilization has been further explored by means of Molecular Dynamics (MD) simulation, either at room temperature or at a high temperature, with the aim of verifying whether the experimental trend observed with variable temperature CD can be reproduced by simulation.

## 2. Results and Discussion

### 2.1. CD Spectra Interaction with c-myc G-Rich DNA

With the aim to shed light on the possible mechanisms underlying the observed anticancer activity of PBTs and, in particular, of 8,9-di-Cl (Table 1) in comparison to some of the other less active compounds of the same series, we evaluated the potential of this derivative in binding c-myc GQ. This nucleic acid belongs to the family of GQ-forming DNAs, structures of biomedical importance as their interaction with drugs can lead to desirable anticancer effects [15,16,17,18]. In fact, c-myc binders are considered potential anticancer drugs as, targeting a promoter GQ present upstream of the proto-oncogene Myc, they may lead to the downregulation of its expression [19].

**Figure 2:**
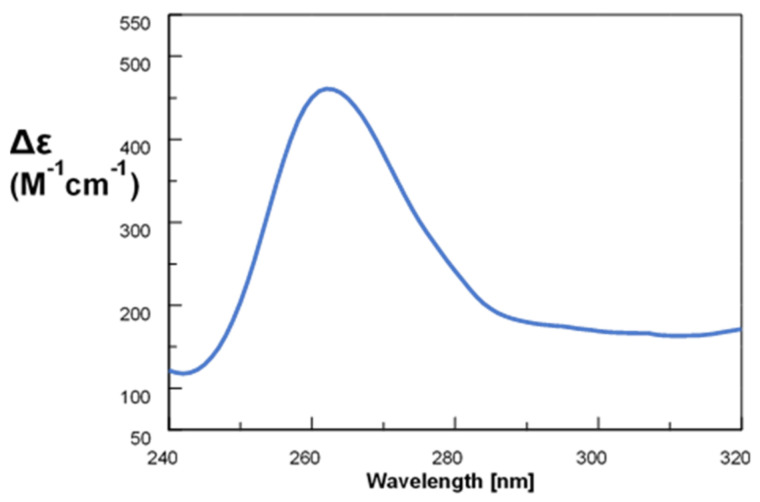
CD spectrum of c-myc 2.5 μM in 1x PBS (T = 40 °C).

In our CD study, we observed a spectrum for c-myc corresponding to a GQ with parallel topology, as identified by the characteristic positive peak at ~265 nm and the negative one at 240 nm (Figure 2) [20,21].

**Figure 3:**
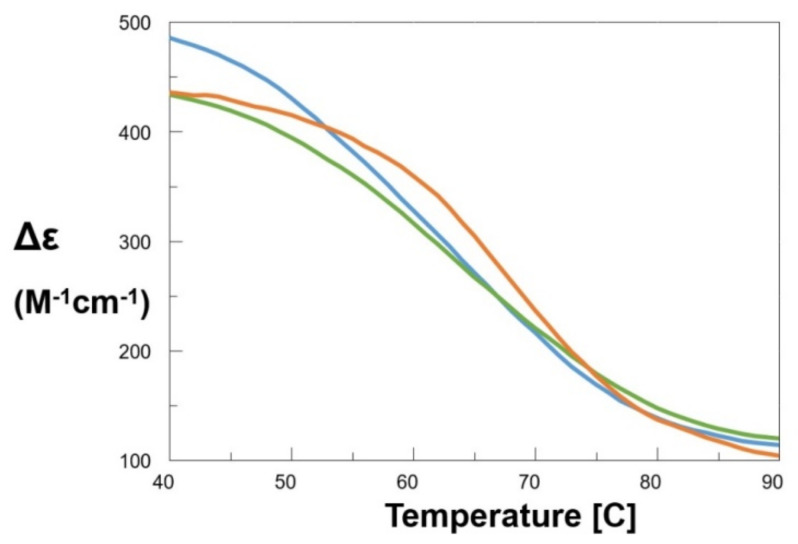
CD denaturation curves of c-myc (green), c-myc+50 equiv. 8-H (azure) and c-myc + 50 equiv. 8,9-di-Cl (orange) in 1x PBS, pH 7.4.

Thus, we studied the effect of 8,9-di-Cl and other closely related compounds (8-Cl, 9-Cl, 8-Me, 8,9-di-Me, 8-CF_3_, 8-CN, and 8-H) on the stability of this GQ DNA by recording, for c-myc and its mixtures with each PBT, the CD values at 265 nm as a function of temperature within the 40–90 °C temperature range (Figure 3 and Figure S1).

In particular, we found a noticeable thermal stabilization for compound 8,9-di-Cl, promptly detectable by the increased value of the melting temperature (T_m_ = 69.2 °C) with respect to the c-myc reference (T_m_ = 65.2 °C), leading to a ΔT_m_ of about +4 °C. By removing the chlorine of position 9 on the PBT scaffold, we reached the 8-Cl derivative. Here, a ΔT_m_ decrease of about 2 °C was observed, i.e., the half caused by the di-chloro-substituted derivative (Table 1). Curiously, no stabilizing effect was found moving the chlorine from position 8 to position 9. Total replacement of the two halogens by H atoms did not provide stabilization. On the contrary, for compound 8-H, a slight DNA destabilization seemed to appear. The same trend was followed by the other compounds 8-CF_3_ and 8-CN, where no GQ DNA T_m_ increase was observed, as shown in Table 1.

Overall, the CD analysis performed on our compounds with c-myc suggested that a C-8 substitution by Cl leads to a significant GQ stabilizing effect. However, in this context, the presence of a further Cl atom in position 9 seemed to reinforce the complex, thereby producing the highest stabilization of the parallel GQ structure. In other terms, C-8 and C-8,9 substitutions by Cl led to effective stabilizers of parallel GQ DNA. Interestingly, the derivatives presenting these Cl substitutions (and in particular 8,9-di-Cl and 8-Cl) were also those endowed with the highest antiproliferative effects on MCF-7, 16-HBE, SW-620, CCRF-CEM, and particularly against COLO-205 (NCI) cancer cells [5]. All the three chlorinated compounds were able to interact with dsDNA, as ascertained by LD studies [6]. On the other hand, CD data suggest that besides their ability to induce significant dsDNA structure perturbation, together with the other biological properties of Table 1, the observed compound-induced antiproliferative activities also correlated with their GQ stabilizing effect. The compound with the highest antiproliferative activity (8,9-di-Cl) also induced the highest c-myc stabilization and arrested the cell cycle in S phase, similarly to other aromatic compounds in the literature that are able to bind GQ DNA and to exert anticancer activity [22,23]. Moreover, we confirmed that our findings are in agreement with those of Li et al. [24], showing that a quinazoline derivative, QPB-15e, inhibited SMMC-7721 liver cancer cell proliferation and stabilized the c-myc GQ.

### 2.2. Docking Results

**Figure 4:**
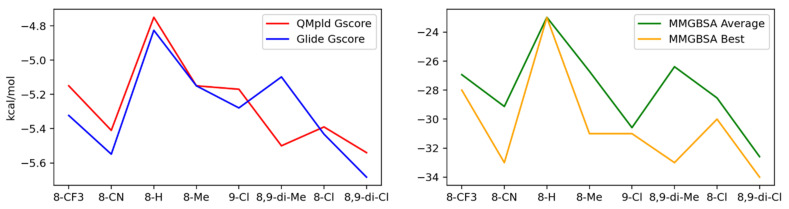
(Left) Best QM-pld G-Score and Glide XP G-Score. (Right) Best and average MMGBSA dG binding free energy.

The flexible docking of the eight selected PBTs compounds was performed with Glide in XP mode using as targets the 20 G-quadruplex conformations comprised in the 1XAV PDB (see Table S1). The results indicate the conformation number 20 as the best target, having the best docking poses with most of the ligands. Data in Table S1 show that some other conformations led to very poor results, or even to no poses at all. Conformation number 20 was then used as the target for the QM-pld procedure (quantum polarized ligand docking), and MMGBSA (molecular mechanics energies combined with generalized born and surface area continuum solvation) dG calculations were performed on the QM-pld poses (see Table S2 for details). Figure 4 shows the best G-Score, obtained with Glide XP and QM-pld, and the best and the average MMGBSA free binding energies obtained for the eight studied ligands. The docking G-Score and MMGBSA binding energies suggest that 8,9-di-Cl is the best candidate, and 8-H the worst. This is in agreement with the quadruplex stabilization observed in the CD experiment for the complex 8,9-di-Cl and with the fact that the compound 8-H is thermally destabilizing the GQ structure. However, either the docking free binding energy order or the G-Score values do not completely agree with the order of the GQ stabilization showed by the CD experiments; 8-CF_3_ and 8-CN have a rather good binding energy according to the docking results, but they are not capable of stabilizing its structure according to the CD results. 8,9-di-Me displays a rather good score considering the best QM-pld and MMGBSA dG bind poses, and experimentally leads to a slight thermal stabilization, as seen with CD experiments, and it is able to inhibit the S phase of the cell cycle as well as the ligand 8,9-di-Cl, as reported in Table 1.

**Figure 5:**
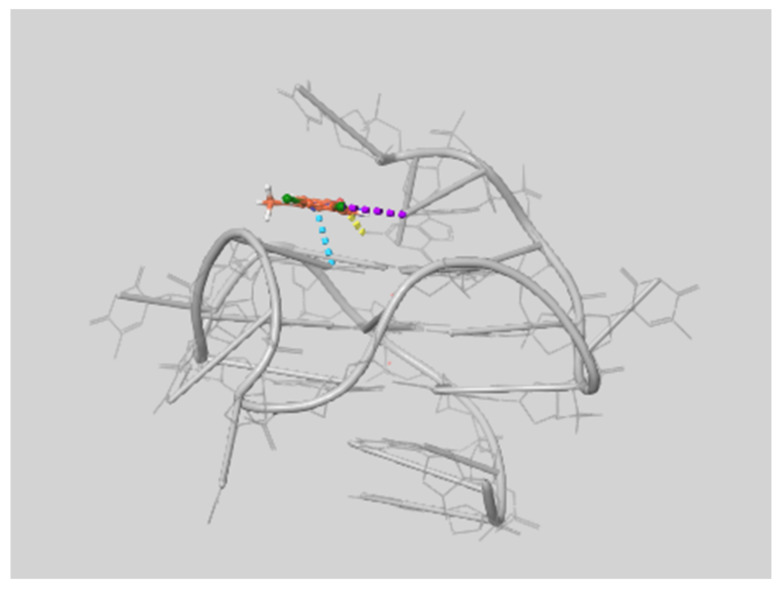
Interaction between the ligand 8,9-di-Cl and c-myc at the 5′ end of the GQ. Dashed lines highlight specific interactions according to the following color code depending on the nature of the interaction: hydrogen bond (yellow), π-π (light blue), and halogen bond (purple). Carbon atoms of the ligand have a brown color, while Hydrogen atoms are white, Oxygen red, Nitrogen blue, Chlorine green, and all atoms on DNA gray.

For all the ligands, the best pose was observed on the 5′ side of the GQ, as exemplified in Figure 5.

**Figure 6:**
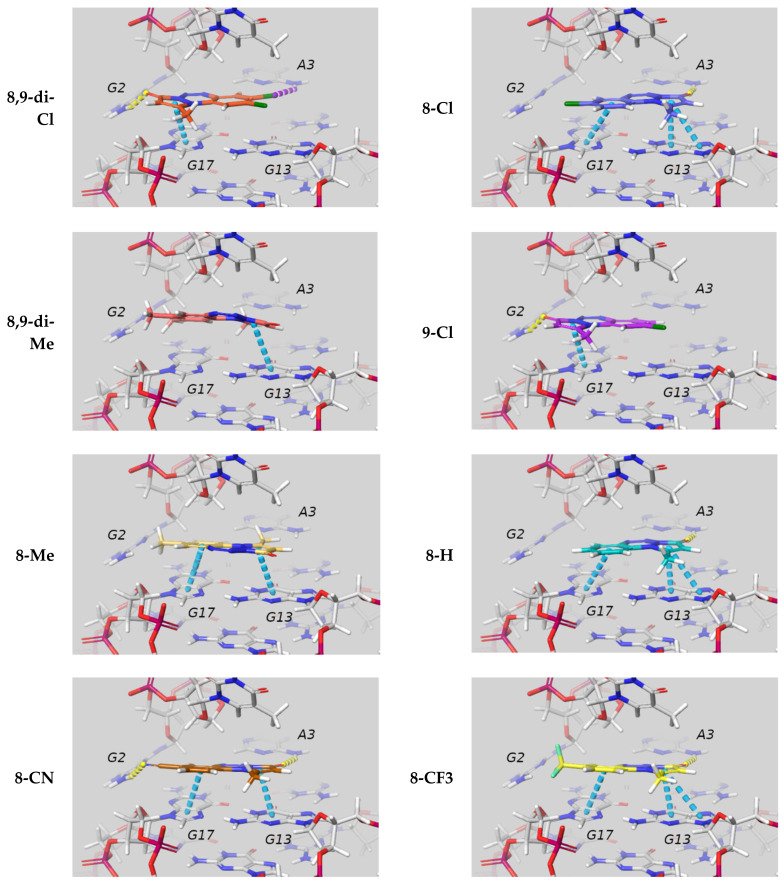
Interaction with the GQ c-myc, in the best pose according to the MMGBSA dG bind. The ligands are all located at the 5′ end of the GQ. Dashed lines highlight specific interactions according to the following color code depending on the nature of the interaction: hydrogen bond (yellow), π-π (light blue), and halogen bond (purple). For artistic purposes, the Carbon atoms of the ligand vary the color with the ligand, while the Hydrogen atoms are white, Oxygen red, Nitrogen blue, Fluorine light green, Chlorine green, and the carbon atoms on DNA are light gray.

By visual inspection of the best docking poses for all the ligands (see Figure 6) we can draw some general conclusions concerning ligand–GQ interactions. As expected, π-π interactions and hydrogen bonds (HBs) both play important roles, and they often involve the residues highlighted in Figure 5: guanine 13 or 17 often interact with the benzene and/or the pyrazolone ring of the ligands; HB are formed between guanine 2 and the oxygen atom of the pyrazolone ring, as well as between adenine 3 and the nitrile in of the 8-CN ligand. For the chlorine derivatives, we observed a halogen bond between adenine 3 and the chlorine atoms.

#### Comparison with Drug-Like Ligands

**Figure 7:**
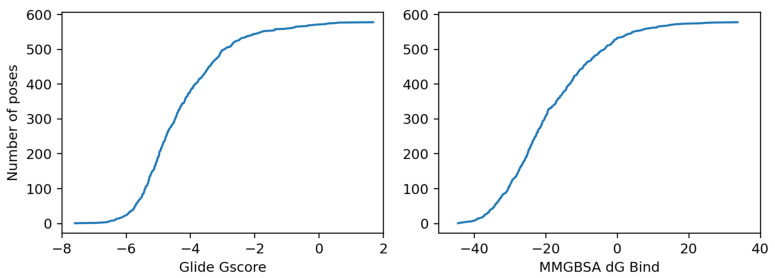
(**left**) Glide G-Score and (**right**) MMGBSA dG binding energy for the poses obtained on a set of ~130 drug-like molecules having a MW close to that of the studied compounds. All values are in kcal/mol.

In this section, we compare the G-Score and the MMGBSA dG values obtained for our compounds with those obtained, in a separate set of calculations, by docking a set of drug-like molecules taken from a database made available by Schrödinger into the 1XAV GQ structure. A molecule with a high affinity for a given target is expected to have higher scores compared to other molecules randomly chosen simply according to the drug-like features. From the Schrödinger database of 2000 drug-like molecules, we selected 130 molecules according to the following criteria: (i) a molecular weight close to that of the studied ligands, between 200 and 269 Da; (ii) not possessing a positive charge, since our ligands have no charge. The selected molecules were docked with Glide XP and about 578 poses were obtained, and the MMGBSA dG values were calculated on those poses. The results are shown in Figure 7.

As shown in Figure 7, the drug-like molecules reached lower values (G-Score < −7 kcal/mol; MMGBSA dG binding < −45 kcal/mol) than our PBT compounds, for which the best G-Score was –5.6 kcal/mol and the best MMGBSA dG was equal to –33.9 kcal/mol.

According to this comparison, our compounds are not expected to be better binders for the GQ compared to random molecules taken from the Schrödinger database. The MD simulations and CD provide a more conclusive analysis as discussed below.

### 2.3. MD Simulations

The anticancer activity of drugs interacting with quadruplexes is related to their capability to stabilize the GQ conformation [24]. A series of Molecular Dynamics simulations were performed to verify whether the complexes obtained through the docking process remain stable in an explicit solvent water solution, and if their formation can stabilize the quadruplex structure.

**Figure 8:**
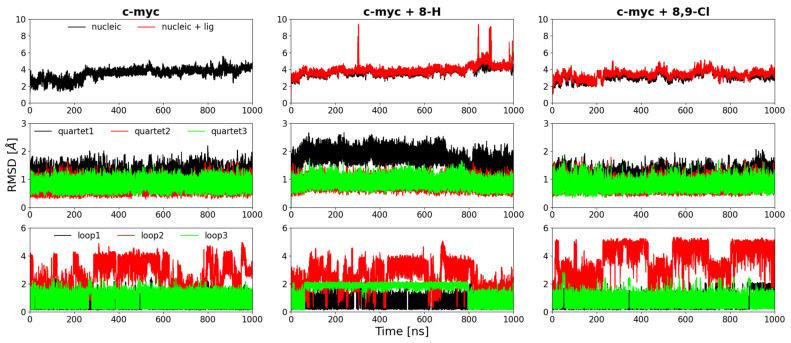
RMSD of the simulated systems at 350 K (NPT). Each column refers to a different system: (left column) c-myc without ligand, (central column) c-myc + ligand 8-H, and (right column) c-myc + ligand 8,9-di-Cl. Each row represents a different set of heavy atoms selected for the RMSD calculation. First row: in black all DNA residues, in red all residues + ligand. Second row: black, red, and green are the first, the second, and the third loop, respectively. Third row: black, red, and green are the first, the second, and the third quartet, respectively. Each simulation is 1000 nanosecond (ns) long.

Root-mean-square deviation (RMSD) calculations were used to analyze the deviations of the quadruplex during the MD trajectories, using the starting structure as a reference. Structural denaturation manifests in an increase of the RMSD. It is known that different regions of the quadruplex, specifically the tetrads and the loops, have different mobilities [25]; therefore, the RMSD was calculated either on all DNA atoms (excluding hydrogen atoms) or on the nucleotides in the tetrads or in the loops region separately, as shown in Figure 8 for the three systems simulated: (i) GQ c-myc without ligands, (ii) the complex of the GQ c-myc and the ligand 8,9-di-Cl, and (iii) the complex GQ c-myc with ligand 8-H. Together with the RMSD of the DNA atoms, we also report the RMSD of the complexes (i.e., c-myc + ligand); the latter RMSD is useful to verify whether the ligand leaves the docking site during the simulations, since when it occurs, the RMSD should significantly increase, see, e.g., the RMSD with the ligand 8-H.

For the ligand-free system, or with the 8-H ligand, the RMSDs in the last part of the simulation were higher than 4 Å, while that with the 8,9-di-Cl ligand was significantly lower. This suggests a stabilizing effect due to the complexation with the 8,9-di-Cl ligand, in agreement with the CD measurements. It is important to note that the CD signal is mainly related to the stability of the relative positions of the guanine tetrads, and that in presence of the 8,9-di-Cl ligand, the RMSDs of the tetrads are the lowest. On the other hand, the complex with the 8-H ligand has higher RMSD compared to the ligand-free quadruplex, in accordance with the destabilizing properties indicated by CD experiments.

While the origin of the larger RMSD of the first tetrad in system 2 is rather elusive and requires further structural analysis that is beyond the scope of the present work, the origin of the concomitant peculiar variation of loop 3 in the same system observed in Figure 8 can be easily interpreted by visual inspection of the trajectory. Indeed, loop 3 is formed by only one base, and in this simulation, it can be either exposed fully to the solvent pointing outside the GQ, as shown in Figure 5, or inserted in the nearby groove, also interacting with the first tetrad. This behavior of loop 3 was seldom observed in the other simulations (see Figures S2–S12), while the destabilization of tetrad 1 in system 2 was observed in most simulations.

The stability of the secondary structure of nucleic acids is strongly related to their interaction with their counterions, to the point that the ions are considered as an integral part of nucleic acids structure and are the subject of extensive experimental and computational studies [25,26,27,28]. The GQ structure is known to be stabilized by monovalent ions in the central channel formed by the quartets. Interaction with alkali ions of nucleic acid in solution can be studied by exploiting the NMR properties of DNA nuclei and/or those of the ions [29,30] to obtain important information on the residence time of the ions in close proximity of DNA. While the residence time of alkali ions at the DNA surface are estimated to be in the ps-μs time scale, the residence time in the GQ channel is much longer, and can even reach 1 h [31]. In particular, for K+ in parallel GQs—as studied in the present work—a residence time of 250 ms has been estimated [32]. Therefore, no ion exchange is typically expected in MD simulations in the microsecond time scale at room temperature. However, it is important to highlight that the correct reproduction of the ion behavior in MD simulations is dependent on the proper choice of ion/water parameters [25,26,33,34]. The used combination of parameters is known to work well for this type of system, and we did not observe any exits of the ions in the simulations up to the temperature of 500 K. At higher temperature, equal or larger than 550 K, we observe that the exit of the ions was often concomitant with the GQ denaturation.

Despite the high simulation temperature none of the systems denatured, because the scale time of this phenomenon in the experiments is much greater than the microsecond, and thus, we cannot expect to see the denaturation in the simulations of the length of 1000 ns simulation.

#### MD at High Temperature

Docking allows for identifying of the best pose for a ligand at the surface of the quadruplex and provides an estimate of the binding free energy.

Ideally, we would like to verify if the stabilizing compounds can really keep the quadruplex structure stable with the MD simulations performed at the potentially denaturing temperature. However, the time required for the denaturation of the “wet sample” would be by far too long to be simulated “in silico” at normal conditions. An approach used in computer simulations to follow the denaturation in a reasonable simulation time takes advantage of the possibility of inducing and speeding up the thermal denaturation process by raising the temperature [35,36,37]. It is important to stress that this approach is possible due to the benefit offered by classical MD using harmonic bonds in the force field, and that the behavior at a high temperature is not expected to correspond to the behavior at such a temperature, but rather to enable speeding up the sampling. Indeed, it has been noted in other cases that increasing the temperature accelerates the protein unfolding without changing the “pathway”, and we therefore assume that the same strategy can be used here to verify the stabilization capability of the quadruplex by the selected ligands.

MD simulations were run at different temperatures: 300 K, 350 K, 500 K, 550 K, and 600 K, either for the GQ alone or with the 8,9-di-Cl ligand or the 8-H ligand (See Section 3.3.3 for details).

Up to a temperature of 500 K, during the simulated time interval of 200 ns (see Figures S2–S12 in the Supplementary Materials) the quadruplex was stable, either alone or with either of the two ligands, indicating that a higher temperature is required to model the denaturation process within the accessible time scale for simulations.

**Figure 9:**
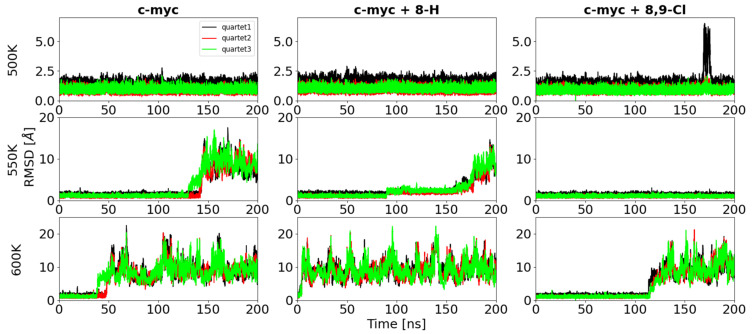
Time evolution of the RMSD with respect to the initial structure in the simulation at: 500 K (top row), 550 K (middle row), and 600 K (bottom row), calculated for the quartets of the ligand-free c-myc (left column), the c-myc + 8-H (central column), and c-myc + 8,9-di-Cl complexes.

The RMSD of the quartets calculated for the three systems at 500 K, 550 K, and 600 K, are respectively reported in Figure 9. It should be noted that each simulation was repeated three times, and the results that we do not show here are reported in the Supplementary Materials in Figures S13 and S14.

Within the simulation time range of 200 ns, the c-myc + 8,9-di-Cl system did not denature, nor at 500 or at 550 K, while both the c-myc alone or in complex with 8-H denatured at the temperature of 550 K. The same behavior was observed when repeating the simulations changing the initial velocity distribution (see Figures S13 and S14).

**Figure 10:**
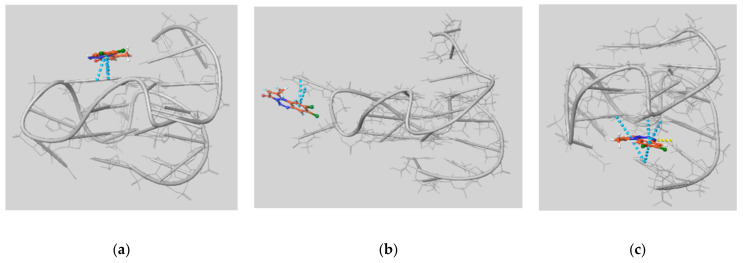
Different modes of π-π (light blue dashed lines) and H-bond (yellow dashed lines) interactions of 8,9-di-Cl with c-myc at 500 K, observed during the migration of the ligand from one docking site to another on the opposite side of the GQ: (**a**) frame at 40 ns: π-π interactions with the guanine 13; (**b**) frame at 45 ns: π-π interactions with adenine 12 belonging to the second loop; (**c**) frame at 50 ns: π-π interactions with guanine 6 and 10 and adenine 21, h-bonding with thymine 20.

It is interesting to note that in most of the simulations at 500, 550, and 600 K, both the 8-H and 8,9-di-Cl ligands moved from the original docked position on the terminal 5′ quartet of the GQ and boundagain on the terminal 3′ quartet (Figure 10), and in some cases, they remained in this docking site until the end of the simulation. As illustrated in Figure 10b, the sliding between the two quartets can be facilitated by interactions with the bases in the loops. Interactions between the loops’ bases and a variety of ligands, such as hemin, pentacyclic acridine compound RHPS4, Telomestatin, porphyrins derivative TMPyP4, benzothiazole-based CX-5461, the tri-substituted acridine BRACO19, Thioflavin T, and other ligands, have already been observed in recent studies by Stadlbauer et al. [38], Mulholland et al. [39], Sullivan et al. [40,41], Machireddy et al. [42,43], and Luo et al. [44].

The binding energy of the complexes shown in Figure 10 is calculated with the software Prime and reported in Table 2.

Overall, the comparison of the results of the simulations with different ligands at high temperatures indicated that the 8,9-di-Cl ligand stabilized the c-myc structure, since the simulation temperature required to observe the denaturation within a few hundred ns was higher than for the c-myc alone or with the 8-H ligand. This result is in good agreement with the CD findings that the 8,9-di-Cl is capable of stabilizing the GQ structure. On the other hand, the 8-H ligand does not have this property, and on the other hand, seemed to destabilize the quartet organization, as shown by the RMSD of the quartets at 300 and 350 K (Figure 8, Figures S2 and S3) and by the lower denaturation temperature observed in the simulations. Additionally, this finding is in very good agreement with the CD results, which indicate that this ligand reduces the melting temperature by few degrees.

### 2.4. Ensemble Docking on Target Conformations Obtained from MD Simulations

From the trajectories at 350 K of the three systems, i.e., (1) ligand-free c-myc, (2) c-myc + 8-H, and (3) c-myc + 8,9-di-Cl, the GQ coordinates were extracted every 100 ns (10 conformations for each trajectory). Further docking was then performed on these GQ structures to verify to which extent the results of the initial docking performed on the NMR structure of the PDB were affected by the employed target structure, which was experimentally obtained in the absence of any ligands. In Table 3 and Table 4, the G-Score and MMGBSA values are reported for the best poses of the eight studied compounds for the three GQ conformations extracted from the MD; however, only a few of these were good targets for the ligand, and in many cases no pose was found; as an example, of the 10 conformations taken from the MD simulations (1), only the docking extracted at 900 ns led to poses for some of the compounds, while no poses were found for the other extracted conformations. Similarly, only a few of the sampled GQ conformations extracted from the MD trajectories performed on the complexes with the ligands were good targets for the PBT compounds.

**Figure 11:**
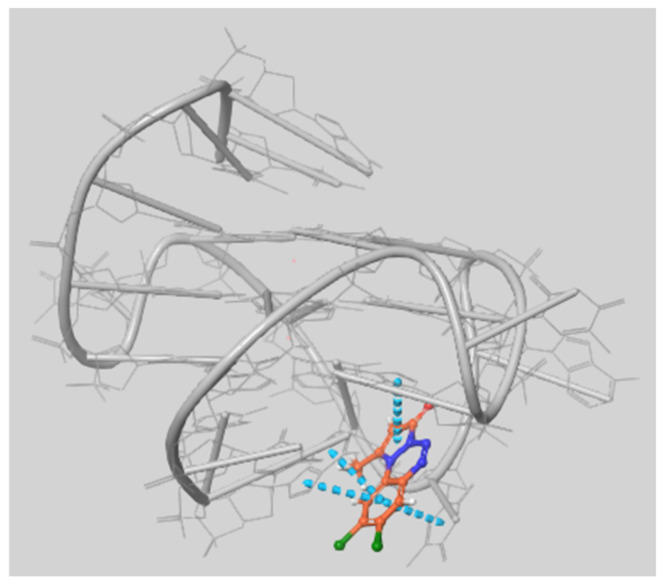
Pose observed at the 3′ end of the quadruplex in the ensemble docking of 8,9-di-Cl on the G-quadruplex conformations extracted from the Molecular Dynamics simulations at 350 K of the system (2).

The conformations extracted from the MD of c-myc with either 8-H or 8,9-di-Cl showed better G-Score values and MMGBSA dG bind values than the conformations extracted from the MD simulations performed without any ligands. Indeed, only 1 out of 10 conformations sampled from the MD trajectory of the system (1), did achieve energetically favorable poses for the ligands, and almost half of the ligands did not have any pose at all. Furthermore, the scoring values obtained were much worse compared to those obtained using the experimental conformations. This could be because during the simulation the quadruplex can adopt conformations that do not allow access of the ligands to the most favorable docking region, since the bases of the loops tend to often “cover” the external quartet when no ligand is present. This tendency may either be overestimated in the simulation, or somehow underrepresented in the experimental NMR structure. On the contrary, the ensemble docking on the conformations extracted from the MD simulations in the presence of the ligands overall improved the value of the docking scores and revealed a new docking site in the 3′ end of the filament, as shown in the Figure 11. On the other hand, the trend of the best MMGBSA dG binding energies or G-Score did not correlate at all with the ligand’s effect on the T_ms_, the exception given for the indication of 8,9-di-Cl as the ligand with the highest binding energy, as was also observed in the docking on the experimental conformations.

## 3. Material and Methods

### 3.1. CD Studies

CD studies were carried out in analogy to other literature studies on similar systems [45]. In particular, we obtained the CD spectra on a J-810 spectro-polarimeter (Jasco Europe S.R.L., Cremella, Italy) equipped with a Peltier PTC-423S/15 temperature controller, using a Hellma (Milan, Italy) quartz cell (0.1 cm). The spectra were measured within the 200–320 nm wavelength range. After normalization for the concentration, the spectra were converted to delta epsilon Δε (M^−1^ cm^−1^) through the use of Equation (1), where θ_λ_ is the observed CD in millidegrees at the given wavelength λ, M is the GQ DNA concentration in molar, and l is path length in cm.

Δε_λ_ = θ_λ_/(32982 × M × l) (1)

In all experiments, we used 2.5 µM of DNA (c-myc, 1 equiv.; Eurofins, Italy) + 125 µM compound (50 equiv.) in 1×PBS (pH 7.4; Sigma Aldrich, Milan, Italy) buffer. All DNA-containing solutions were annealed at 95 °C for 5 min and left overnight at room temperature to slowly cool down (16 h). The presented melting curves (obtained recording Δε_265nm_ *vs* T in the 40–90 °C temperature range) are the average of the three experiments. Melting temperature (T_m_) values were determined as the temperatures relative to minima of the 1st derivative plots of the denaturation curves of Figure 3 and Figure S1. All experiments were repeated at least three times and all spectra were recorded in triplicate.

### 3.2. Docking

**Figure 12:**
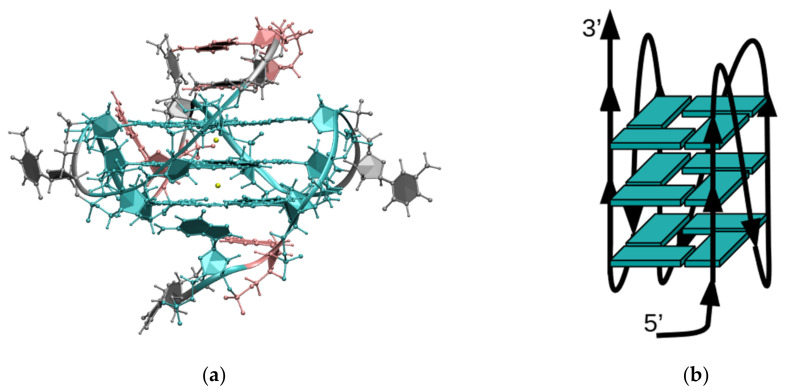
(**a**) Ball and stick representation of the 20th set of coordinates of 1XAV, representing the residues with the following color code: cyan, guanine; pink, adenine; gray, thymine; yellow, potassium. (**b**) Schematic representation of the full parallel-type monomolecular structure.

The study of the interaction between the GQ and the ligands was carried out by using the Schrödinger suite (v. 2016) [46]. As a receptor model we used the three-dimensional NMR solution structure of c-myc G-quadruplex, retrieved from the Protein Data Bank with the code: 1XAV [47]; 1XAV comprises 20 sets of coordinates and has the same topology and sequence as the GQ analyzed with the CD studies. The adopted topology has three G-tetrads organized in a full-parallel G-strands arrangement, with three propeller loops on the side of the quadruple helix; Figure 12 shows a schematic representation of the topology of the 1XAV structure.

The size of the docking grid box was made large enough to include the full quadruplex (30 × 30 × 30 Å). The ligands were prepared using the software 2D-Sketcher [46] and LigPrep [48] from Schrödinger suite. OPLS3 [49] force field was used for both the ligands and the receptor.

The following docking methodologies were employed:

(i) Flexible ligand ensemble docking was performed with Glide [50,51,52] considering the target receptor as rigid. All the 20 conformations, comprised in the PDB 1XAV entry, were used in the calculations as target. Other target conformations were obtained from MD simulations at 350 K, performed in presence and in the absence of ligands. See below for details of the MD simulations.

(ii) Quantum polarized ligand docking calculations [53,54] were performed on the poses obtained from the docking in step (i), using as the target the conformer number 20, which showed the best interaction energy for most of the ligands in step (i) (see SI, Table S2).

(iii) Ligand binding free energies were calculated on the complexes with the poses obtained in step (ii), using the MMGBSA (molecular mechanics energies combined with generalized born and surface area continuum solvation) method in the software Prime [55,56]. The different poses of the ligands in the complex were ranked using the Glide scoring function G-Score [51,52] and MMGBSA dG binding free energies [56].

The results were compared with those on drug-like compounds selected from the Schrödinger database, which had a comparable molecular weight to that of the studied compounds (in the range of 200 and 269 Da).

### 3.3. Molecular Dynamics Simulations

MD simulations were performed to generate an additional ensemble of conformations to be used in the docking procedure and to control the effect of the ligand on the quadruplex’s melting temperature.

As a starting geometry for MD simulations in water solution we used the following: (i) the NMR structure 1XAV, selecting the conformer number 20; and (ii) the same quadruplex structure with the best docking pose with the ligand 8,9-di-Cl or 8-H.

#### 3.3.1. Force Field Parameters

One of the most important choices when setting up a nucleic acid MD simulation is the choice of the force field including the solvent and ion parameters [25,26,33,34,57,58]. Simulations performed in the last two decades have highlighted the importance of proper tuning of the dihedral parameters of the nucleic acid backbone, both for B-DNA and for unconventional nucleic acid conformations such as Z-DNA or quadruplexes [57,58,59,60,61,62]. For non-canonical DNA, the OL15 [60] version of the amber force field is suggested [57]. This version is based on the parm99 version of the Cornell et al. force field [63] with a refinement of the sugar-phosphate backbone torsional angles [59,60,61,62]. Therefore, we used the OL15 force field for the DNA in our simulation.

The libraries with the force field (FF) parameters for the ligands were prepared using the Antechamber package [64] with a standard procedure, which comprises the following steps. The ligand structure optimization was performed using the Gaussian 09 [65] software at HF/6-31G(d) level and the atomic charges were calculated using the Antechamber Restrained Electrostatic Potential Atomic (RESP) fitting procedure [66]; then, the residue libraries with the Generalized Amber Force Field (GAFF) parameters [67] were created with the Antechamber program. Considering previous studies that showed how the GQs’ MD simulation results can be highly affected by the choice of the water model and of the ion parameters, the SPC/E [68] model was used for water molecules, together with the Joung and Cheatham parameters for the K+ ions, and optimized for the SPC/E model [69,70].

#### 3.3.2. Initial Configurations

The initial simulation configuration was set up using the TLEAP module of the AMBER (both version 16 and 18) simulation package. The GQ, alone or in the complex, was surrounded by about 5.5 * 10^3^ water molecules. Potassium cations were added to neutralize the net negative charge of the nucleic acid, two of them being initially placed inside the channel as in the PDB structure, and enough KCl was added to reach the physiological salt concentration of 150 mM (total number of ions: 41 K^+^ and 20 Cl^−^).

#### 3.3.3. Equilibration and Production

The system was equilibrated using a sequence of energy minimizations and constant volume Molecular Dynamics starting from a low temperature and gradually raising it to the target temperature during the initial 60 ps of MD simulations. The final part of the equilibration was performed at constant temperature and pressure, and the production was carried out at constant temperature and volume (NVT ensemble). One set of simulations, at 350 K, was also performed at a constant temperature and pressure (NPT ensemble). The equilibration was performed using the SANDER and Particle Mesh Ewald Molecular Dynamics (PMEMD) modules [71,72,73]; only the latter module was used for the production. The MD simulations were performed at 5 different temperatures (300, 350, 500, 550, and 600 K), which were maintained using the Berendsen thermostat/barostat [74], with a 10 ps time constant for the heat coupling. Simulations at the high temperature were repeated 3 times each (as reported in Table 5), changing the random seed at the beginning of the production run.

Bonds involving hydrogens were constrained using the SHAKE algorithm [75]. Electrostatic interactions were calculated by the particle mesh Ewald method [76] as implemented in AMBER 16, with a cubic B-spline interpolation order and 0.00001 tolerance for the direct space sum cutoff. The time step was set to 2 fs for the room temperature simulation, and 1 fs for the higher temperature, considering the increase of the amplitude of bonds vibration with the temperature. The coordinates were saved every 5 ps. In total, approximately 12 microseconds of MD simulations were performed.

We note that, while the force field parameters for biomolecules are generally expected to be valid at room temperature, there is a large number of studies [35,36,37] showing how the same parameters can be used successfully to perform the simulations at a much higher temperature, as the one used in the present study.

#### 3.3.4. Analysis

The root-mean-square deviations (RMSD) of the atomic positions were calculated using the CPPTRAJ [77] program.

### 3.4. Compounds Synthesis

Synthesis of the compounds was achieved according to the procedure described in previous works [4,5]. The substituents in the PBTs studied in the present work possess different electronic characteristics: poor electron-donating groups (8-Me, 8,9-di-Me), electron-withdrawing groups (8-Cl, 9-Cl, 8,9-di-Cl, 8-CN, 8-CF_3_), or neutral groups (8-H).

## 4. Conclusions

By a combination of in silico (docking and MD simulations) and experimental approaches (CD thermal denaturation), we verified that some tetrazines with selected substituents are capable of slightly stabilizing a c-myc GQ structure. Their anticancer activity [4,5], previously observed in vitro, correlates with this stabilizing action. Indeed, the trend in antiproliferative activities observed in several cellular lines, and especially in MCF-7, 16HBE, COLO-205, SW-620, and CCRF-CEM, can be correlated with the melting temperature observed by CD. This stabilization effect can act together with the mechanisms of action already proposed [4,5,6].

According to the presented docking studies performed on the experimental structures of the considered GQ, the chosen ligands are all able to interact with the c-myc GQ. Furthermore, the compound with a higher affinity according to the docking (8,9-di-Cl) was also the compound leading to the highest stabilization in the CD experiments. However, the docking on the NMR experimental GQ conformations, obtained in the absence of any ligands, led to ligand poses only on the 5′ side of the GQ. Ensemble docking on the GQ structures extracted from a MD simulation of c-myc in an aqueous solution at 350 K, without any ligand, did lead to a reduction of the number of poses for all the ligands with respect to those obtained using the experimental conformations as target, and for many ligands it led to no poses at all. In addition, it also led to worse scoring values (both G-Score and MMGBSA dG binding energy). On the contrary, the ensemble docking on the GQ conformations extracted from the MD simulations in the presence of the ligands did lead to poses that could not be observed using the experimental conformations as the target, and importantly shows that the ligand can bind on both sides of the quadruplex. This result indicates that, to obtain a good picture of the possible poses of a ligand on GQs, it is important to employ a target generated from MD simulations in the presence of a ligand.

It should be noted that the compounds with a destabilizing effect also had a negative MMGBSA dG binding free energy, according to the docking protocols employed here, with values in the 26–32 kcal/mol range. Overall, with the exception for the most stabilizing compound, 8,9-di-Cl, the docking scores were not correlated with the thermal stabilization capability of the ligands experimentally determined.

Molecular dynamics simulations performed either at 300 K or at higher temperatures up to 600 K on the solvated c-myc, or on its complexes with 8,9-di-Cl or 8-H, are in accordance with the CD data. In particular, in the MD simulations at 300 or 350 K, we observed a greater stabilization of tetrads in the presence of the stabilizing ligand 8,9-di-Cl and a destabilization in the presence of the 8-H ligand, in comparison with the GQ without any ligands. The MD simulations at high temperatures (500, 550, and 600 K) qualitatively reproduced the effect on the melting temperatures of the simulated ligands, with the observed denaturation temperature decreasing in the simulations according to the following order: c-myc + 8,9-di-Cl > ligand-free c-myc ~ c-myc + 8-H.

The MD results in this study are particularly important when considering that the docking study indicates that both the stabilizing and destabilizing compounds display a similar negative binding free energy, while the MD simulations discriminate the stabilizing/destabilizing activity of the ligands.

While the detailed reason for the different stabilizing effects of the studied compounds is to be analyzed in a future study, we believe that the computational approach proposed here, based on MD simulations at high temperatures, appears extremely promising for verifying, and possibly predicting, the GQ stabilization effect for other classes of compounds and could be employed in future GQ-targeting drug design.

## Figures and Tables

**Table 1:** Effects PBTs on c-myc melting temperature (T_m_), linear dichroism (LD) data on duplex DNA (ΔLD_260nm_) ^b^, and other biological properties. Compounds are listed following the thermal stability order, starting from the most stable.

Compounds	T_m_ (ΔT_m_)/°C ± 0.2 ^a^	ΔLD_260nm_/deg ^b^	TopoII-Inhib.^c^	Cell Cycle ^d^	Apoptosis ^e^ EC_50_ µM	MCF-7 ^f^ IC_50_/µM	16-HBE ^f^ IC_50_/µM
8,9-di-Cl	69.2 (+4.0)	0.151	Y	S Phase	2–4	13.9	13,5
8-Cl	67.1 (+1.9)	0.118	N	-	4–8	20.5	17.7
8,9-di-Me	65.3 (+0.1)	0.025	N	S Phase	3–5	46.7	38.9
9-Cl	65.1 (−0.1)	0.203	Y	-	4–10	29.5	27.5
8-Me	64.2 (−1.0)	0	N	-	3–5	42.6	36.3
8-H	63.2 (−2.0)	-	-	-	-	54.9	ND
8-CN	63.1 (−2.1)	-	-	-	-	35.9	46.7
8-CF_3_	63.0 (−2.2)	-	-	-	2–3	>100	30.9

^a^ All CD experiments were performed in the PBS buffer at pH 7.4. (C_cpd_ = 125 µM; C_G4DNA_ = 2.5 µM; T_m_ for c-myc alone: 65.2 ± 0.2 °C); ΔT_m_ = T_m_Cpd − T_m_c-myc; ^b^ From normalized LD spectral data recorded using salmon testes DNA with (cpd/DNA) = 0 and 0.08 as reported by Lauria et al. [6]; ^c^ Inhibition of the catalytic cycle of topoisomerase II, only selected compounds were tested [6]; ^d^ Cell cycle arrest against HeLa, only the selected compounds were tested [5]; ^e^ Range of apoptosis induction (µM) by mitochondrial depolarization assay [5]; ^f^ [5].

**Table 2:** Prime MMGBSA dG binding energies for the different binding modes represented in Figure 10. Time is expressed in nanosecond (ns).

Time (ns)	MMGBSA dG Bind (kcal/mol)
40	−19.06
45	−17.16
50	−32.75

**Table 3:** Glide XP docking G-Score values expressed in kcal/mol obtained on the Q-quadruplex conformations extracted from the Molecular Dynamics simulations of c-myc at 350 K, or its complexes with the ligand 8-H or 8,9-di-Cl. A “-” sign indicates that no pose was found.

MD Source	Time (ns)	8-CF_3_	8-CN	8-H	8-Me	9-Cl	8.9-di-Me	8-Cl	8,9-di-Cl
(1) c-myc	900	−1.90	−1.58	-	-	-	-	−1.83	−2.01
(2) c-myc + 8-H	600	-	-	−0.27	−0.15	-	-	-	-
	900	−4.46	−1,13	−4.59	−3.87	−4.24	−3.98	−4.12	−4.60
	1000	-	−3.74	-	-	-	-	−4.15	−5.11
(3) c-myc + 8,9-di-Cl	300	−5.21	−5.05	−4.92	−5.80	−5.26	−5.50	−5.09	−5.40
	900	−4.82	−4.69	−4.78	−4.82	−4.59	−5.20	−5.38	−4.97
	1000	−3.09	−3.14	−3.14	−3.36	−3.28	−3.19	−3.09	−3.10

**Table 4:** Prime MMGBSA dG bind values expressed in kcal/mol on the G-quadruplex conformations extracted from the Molecular Dynamics simulations of c-myc at 350 K, or its complexes with the ligand 8-H or 8,9-di-Cl. A “-” sign indicates that no pose was found.

MD Source	Time (ns)	8-CF_3_	8-CN	8-H	8-Me	9-Cl	8.9-di-Me	8-Cl	8,9-di-Cl
(1) c-myc	900	−14.94	−22.23	-	-	-	-	−16.80	−15.90
(2) c-myc + 8-H	600	-	-	−3.69	0,06	-	-	-	-
	900	−26.68	−8.55	−30.57	−29.03	−32.12	−22.08	−32.94	−30.12
	1000	-	−26.83	-	-	-	-	−26.95	−37.11
(3) c-myc + 8,9-di-Cl	300	−27.17	−29.26	−25.20	−25.79	−27.63	−26.48	−29.10	−34.06
	900	−20.71	−24.27	−18.22	−19.56	−25.07	−20.44	−30.89	−26.42
	1000	−15.47	−16.23	−17.44	−20.25	−14.34	−17.79	−21.50	−12.80

**Table 5:** Production length and ensemble conditions of simulations at different temperatures.

Temp. (K)	Production Length (ns)	Ensemble
300	1000	NVT
350	1000	NPT
350	200	NVT
500	(sim. 1) 200 (sim. 2) 200 (sim. 3) 200	NVT
550	(sim. 1) 200 (sim. 2) 200 (sim. 3) 200	NVT
600	(sim. 1) 200 (sim. 2) 200 (sim. 3) 200	NVT

## Data Availability

Data sharing not applicable.

## Supplementary Materials

The following are available online at https://www.mdpi.com/article/10.3390/ijms22116028/s1, Figure S1: CD denaturation curves, Table S1: G-Score values of ensemble docking, Table S2: G-Score, QM-pld protocol, and MMGBSA dG binding energies, Figures S2–S12: RMSD of simulations at 300, 350 (NVT), 500 (3 simulations), 550 (3 simulations), and 600 K (3 simulations), Figures S13 and S14: RMSD of the quartets at 500, 550, and 600 K (simulation n.2 and n.3).

## Abbreviations

16HBE	Human Bronchial Epithelial Cells
CCRF-CEM	T Lymphoblastoid Cells
COLO-205	Colon Cancer Cells
MCF-7	Michigan Cancer Foundation-7 Human Breast Cancer Cell
PBS	Phosphate-buffered saline
SW-620	Human Colon Carcinoma Cells
