# Supplementary material for: Spectroscopic and In Silico Studies on the Interaction of Substituted Pyrazolo[1,2-a]benzo[1,2,3,4]tetrazine-3-one Derivatives with c-Myc G4-DNA
Source: Int J Mol Sci. 2021 Jun 2;22(11):6028. doi: 10.3390/ijms22116028 (PMC8199725; doi:10.3390/ijms22116028)
Supplement: Supplementary file 1 [file ijms-22-06028-s001.zip › ijms-1231735-supplementary.pdf]

International Journal of *Molecular Sciences*

MDPI

Supporting Information

Spectroscopic and In Silico Studies on the Interaction of Substituted Pyrazolo[1,2-a]benzo[1,2,3,4]tetrazine-3-one Derivatives with c-Myc G4-DNA

Simone Mulliri[†,1], Aatto Laaksonen[2,3,4,5,*], Pietro Spanu[6], Riccardo Farris[1], Matteo Farci[1], Francesco Mingoia[7], Giovanni N. Roviello[†,8,*], Francesca Mocci[1,4,*]

[1] Department of Chemical and Geological Sciences, University of Cagliari, I-09042 Monserrato, Italy; mulliri.simone@hotmail.com (S.M); farrisric@outlook.com (R.F); farcifarci@live.it (M.F.) fmocci@unica.it (F.M)
[2] State Key Laboratory of Materials-Oriented and Chemical Engineering, Nanjing Tech University, 210009 Nanjing, China; aatto.laaksonen@mmk.su.se
[3] Division of Physical Chemistry, Department of Materials and Environmental Chemistry, Arrhenius Laboratory, Stockholm University, 10691 Stockholm, Sweden
[4] Centre of Advanced Research in Bionanoconjugates and Biopolymers, Petru Poni Institute of Macromolecular Chemistry, Iasi, 700487, Romania
[5] Department of Engineering Sciences and Mathematics, Division of Energy Science, Luleå University of Technology, SE-97187 Luleå, Sweden
[6] Istituto di Chimica Biomolecolare, ICB - CNR -Trav. La Crucca 3; 07100 -Sassari, Italy; pietro.spanu@cnr.it
[7] Istituto per lo Studio dei Materiali Nanostrutturati ISMN - CNR; Via U. La Malfa 153; I-90146 Palermo, Italy; francesco.mingoia@ismn.cnr.it
[8] Istituto di Biostrutture e Bioimmagini, IBB - CNR; Via Mezzocannone 16; I-80134 Naples, Italy; giroviel@unina.it
*Correspondences: A.L., aatto.laaksonen@mmk.su.se; Tel.: +46-08162372; G.N.R, giroviel@unina.it, Tel: +39-812534585; F.M., fmocci@unica.it, Tel: +39-0706754390
[†] These authors equally contributed to this work

Contents

CD spectra. Interaction with c-myc oncogene promoter 4

Figure S1. CD denaturation curves of c-myc (2.5 µM, ———) and c-myc+50 equiv. of 8,9-di-Me (——), 8-CF3 (———), 8-CN (———), 8-H (———), 8-Me (———) and 9-Cl (———) in 1x PBS, pH 7.4. . 4

Docking results 5

Table S1. G-Score values for the glide XP best poses of ensemble docking for each of the 20 set of coordinates comprised in the PDB file 1xav.pdb. Values are shown in kcal/mol. The used color code varies from green to red ongoing from the best to the worst values; np = no poses 5

Table S2. Best G-Score (Glide XP and QM-pld protocol) and best MMGBSA dG binding energies found on conformation n.20 of 1xav.pdb. The used color code varies from green to red ongoing from the best to the worst values (kcal/mol). 5

MD at high temperature 6

Figure S2. RMSD at 300 K. Each column refers to a different system: (left column) c-myc without ligand, (central column) c-myc + ligand 8-H and (right column) c-myc + ligand 8,9-di-Cl. Each row represents a different set of heavy atoms selection for the RMSD calculation: First row: in black all DNA residues, in red all residues + ligand. Second row: black, red, and green are the

first, the second and the third loop, respectively. Third row: black, red, and green are the first, the second and the third quartet, respectively......6

Figure S3. RMSD at 350 K (NVT). Each column refers to a different system: (left column) c-myc without ligand, (central column) c-myc + ligand 8-H and (right column) c-myc + ligand 8,9-di-Cl. Each row represents a different set of heavy atoms selection for the RMSD calculation: First row: in black all DNA residues, in red all residues + ligand. Second row: black, red, and green are the first, the second and the third loop, respectively. Third row: black, red, and green are the first, the second and the third quartet, respectively......6

Figure S4. RMSD at 500 K. (sim. 1). Each column refers to a different system: (left column) c-myc without ligand, (central column) c-myc + ligand 8-H and (right column) c-myc + ligand 8,9-di-Cl. Each row represents a different set of heavy atoms selection for the RMSD calculation: First row: in black all DNA residues, in red all residues + ligand. Second row: black, red, and green are the first, the second and the third loop, respectively. Third row: black, red, and green are the first, the second and the third quartet, respectively......7

Figure S5. RMSD at 500 K. (sim. 2). Each column refers to a different system: (left column) c-myc without ligand, (central column) c-myc + ligand 8-H and (right column) c-myc + ligand 8,9-di-Cl. Each row represents a different set of heavy atoms selection for the RMSD calculation: First row: in black all DNA residues, in red all residues + ligand. Second row: black, red, and green are the first, the second and the third loop, respectively. Third row: black, red, and green are the first, the second and the third quartet, respectively......7

Figure S6. RMSD at 500 K. (sim. 3). Each column refers to a different system: (left column) c-myc without ligand, (central column) c-myc + ligand 8-H and (right column) c-myc + ligand 8,9-di-Cl. Each row represents a different set of heavy atoms selection for the RMSD calculation: First row: in black all DNA residues, in red all residues + ligand. Second row: black, red, and green are the first, the second and the third loop, respectively. Third row: black, red, and green are the first, the second and the third quartet, respectively......8

Figure S7. RMSD at 550K. (sim. 1). Each column refers to a different system: (left column) c-myc without ligand, (central column) c-myc + ligand 8-H and (right column) c-myc + ligand 8,9-di-Cl. Each row represents a different set of heavy atoms selection for the RMSD calculation: First row: in black all DNA residues, in red all residues + ligand. Second row: black, red, and green are the first, the second and the third loop, respectively. Third row: black, red, and green are the first, the second and the third quartet, respectively......8

Figure S8. RMSD at 550 K. (sim. 2). Each column refers to a different system: (left column) c-myc without ligand, (central column) c-myc + ligand 8-H and (right column) c-myc + ligand 8,9-di-Cl. Each row represents a different set of heavy atoms selection for the RMSD calculation: First row: in black all DNA residues, in red all residues + ligand. Second row: black, red, and green are the first, the second and the third loop, respectively. Third row: black, red, and green are the first, the second and the third quartet, respectively......9

Figure S9. RMSD at 550 K. (sim. 3). Each column refers to a different system: (left column) c-myc without ligand, (central column) c-myc + ligand 8-H and (right column) c-myc + ligand 8,9-di-Cl. Each row represents a different set of heavy atoms selection for the RMSD calculation: First row: in black all DNA residues, in red all residues + ligand. Second row: black, red, and green are the first, the second and the third loop, respectively. Third row: black, red, and green are the first, the second and the third quartet, respectively......9

Figure S10. RMSD at 600 K. (sim. 1). Each column refers to a different system: (left column) c-myc without ligand, (central column) c-myc + ligand 8-H and (right column) c-myc + ligand 8,9-di-Cl. Each row represents a different set of heavy atoms selection for the RMSD calculation: First row: in black all DNA residues, in red all residues + ligand. Second row: black, red, and green are the first, the second and the third loop, respectively. Third row: black, red, and green are the first, the second and the third quartet, respectively. 10

Figure S11. RMSD at 600 K. (sim. 2). Each column refers to a different system: (left column) c-myc without ligand, (central column) c-myc + ligand 8-H and (right column) c-myc + ligand 8,9-di-Cl. Each row represents a different set of heavy atoms selection for the RMSD calculation: First row: in black all DNA residues, in red all residues + ligand. Second row: black, red, and green are the first, the second and the third loop, respectively. Third row: black, red, and green are the first, the second and the third quartet, respectively. 10

Figure S12. RMSD at 600 K. (sim. 3). Each column refers to a different system: (left column) c-myc without ligand, (central column) c-myc + ligand 8-H and (right column) c-myc + ligand 8,9-di-Cl. Each row represents a different set of heavy atoms selection for the RMSD calculation: First row: in black all DNA residues, in red all residues + ligand. Second row: black, red, and green are the first, the second and the third loop, respectively. Third row: black, red, and green are the first, the second and the third quartet, respectively. 11

Figure S13. RMSD of the quartets at 500, 550 and 600 K. (sim. 2). Time evolution of the RMSD with respect to the initial structure in the simulation at: 500 K (top row); 550 K (middle row) and 600 K (bottom row), calculated for the quartets of the ligand-free c-Myc (left column), the c-Myc + 8-H (central column) and c-Myc + 8,9-di-Cl complexes. 11

Figure S14. RMSD of the quartets at 500, 550 and 600 K. (sim. 3). Time evolution of the RMSD with respect to the initial structure in the simulation at: 500 K (top row); 550 K (middle row) and 600 K (bottom row), calculated for the quartets of the ligand-free c-Myc (left column), the c-Myc + 8-H (central column) and c-Myc + 8,9-di-Cl complexes. 12

CD spectra. Interaction with c-myc oncogene promoter

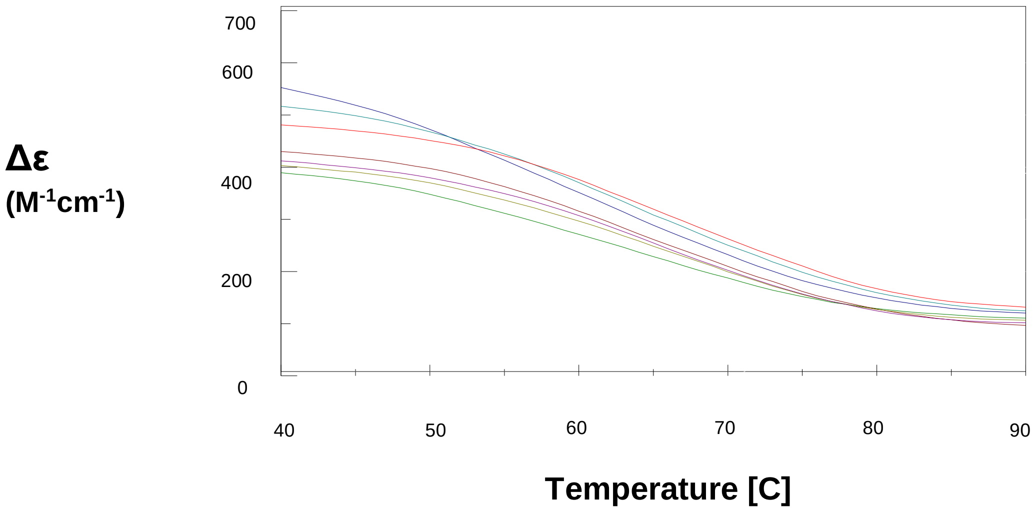

Figure S1. CD denaturation curves of c-myc (2.5 μM, ———) and c-myc+50 equiv. of 8,9-di-Me (——), 8-CF3 (———), 8-CN (————), 8-H (———), 8-Me (——) and 9-Cl (———) in 1x PBS, pH 7.4.

Docking results

Table S1. G-Score values for the glide XP best poses of ensemble docking for each of the 20 set of coordinates comprised in the PDB file 1xav.pdb. Values are shown in kcal/mol. The used color code varies from green to red ongoing from the best to the worst values; np = no poses

1xav PDB	8-CF3	8-CN	8-H	8-Me	9-Cl	8.9-di-Me	8-Cl	8,9-di-Cl
1	-3,498	-3,447	-3,544	-3,687	-3,271	-2,448	-3,222	-3,321
2	-3,415	np	-4,397	-2,821	-3,451	np	-3,628	-3,320
3	-3,230	np	-2,819	-3,137	-3,886	np	-2,817	-3,291
4	-4,973	-4,477	-4,479	-4,727	-4,972	np	-4,439	-4,833
5	-3,987	-3,879	-4,467	np	-4,496	-3,973	-4,203	-4,297
6	-5,319	-4,888	-4,818	-5,327	-4,641	-4,310	-5,149	-5,246
7	-3,951	-1,818	-3,886	-4,175	-4,423	-3,810	-4,096	-4,162
8	np	np	-4,556	np	-4,868	np	np	np
9	-3,527	-2,782	np	np	-3,551	np	-4,076	np
10	-2,618	-1,813	-4,583	-4,324	-2,504	np	-2,688	-3,045
11	-3,420	-3,323	-3,838	-3,771	-3,897	-3,525	-3,910	-4,137
12	-3,270	-2,893	-3,428	np	-4,450	np	np	np
13	-3,963	np	-4,426	-3,595	-4,137	np	-3,443	-4,045
14	-4,734	-2,557	-4,289	-4,427	-4,491	np	-4,478	-4,236
15	-3,434	-4,269	-4,361	-3,598	-4,307	np	-4,257	-3,926
16	-4,305	-1,448	-4,552	-4,129	-4,315	-3,660	-3,912	-4,610
17	-3,635	np	-3,684	np	-4,245	np	-4,036	-5,133
18	-5,079	-5,399	-4,077	-4,095	-4,320	np	-4,184	-4,472
19	-3,598	np	-3,552	-2,967	-3,848	-2,882	-2,790	-3,731
20	-5,323	-5,549	-4,826	-5,150	-5,279	-5,098	-5,432	-5,683

Table S2. Best G-Score (Glide XP and QM-pld protocol) and best MMGBSA dG binding energies found on conformation n.20 of 1xav.pdb. The used color code varies from green to red ongoing from the best to the worst values (kcal/mol).

Cmpd	Glide XP gscore	QM-pld gscore	Prime MMGBSA ΔG Bind
8,9-di-Cl	-5,609	-5,536	-33,881
8-Cl	-5,218	-5,388	-29,940
8,9-di-Me	-5,176	-5,495	-32,566
9-Cl	-5,257	-5,166	-30,857
8-Me	-5,144	-5,148	-30,684
8-H	-4,829	-4,754	-23,090
8-CN	-4,737	-5,412	-32,540
8-CF3	-5,330	-5,153	-28,020

MD at high temperature

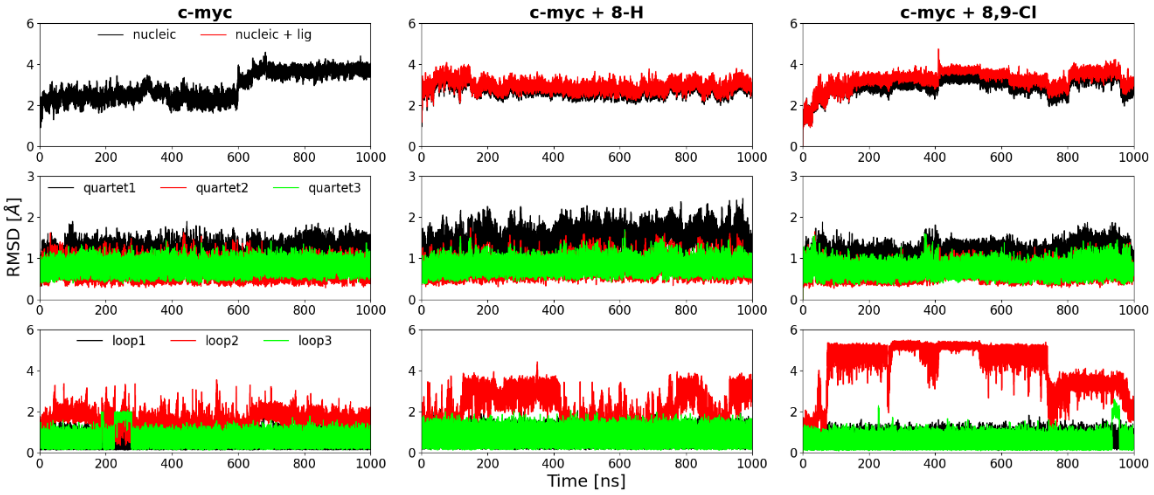

Figure S2. RMSD at 300 K. Each column refers to a different system: (left column) c-myc without ligand, (central column) c-myc + ligand 8-H and (right column) c-myc + ligand 8,9-di-Cl. Each row represents a different set of heavy atoms selection for the RMSD calculation: First row: in black all DNA residues, in red all residues + ligand. Second row: black, red, and green are the first, the second and the third loop, respectively. Third row: black, red, and green are the first, the second and the third quartet, respectively.

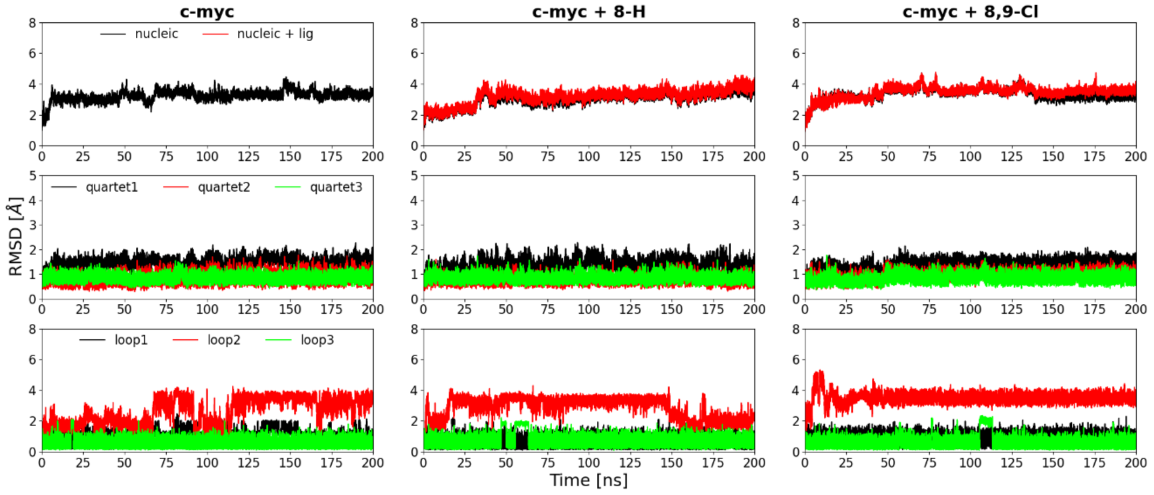

Figure S3. RMSD at 350 K (NVT). Each column refers to a different system: (left column) c-myc without ligand, (central column) c-myc + ligand 8-H and (right column) c-myc + ligand 8,9-di-Cl. Each row represents a different set of heavy atoms selection for the RMSD calculation: First row: in black all DNA residues, in red all residues + ligand. Second row: black, red, and green are the first, the second and the third loop, respectively. Third row: black, red, and green are the first, the second and the third quartet, respectively.

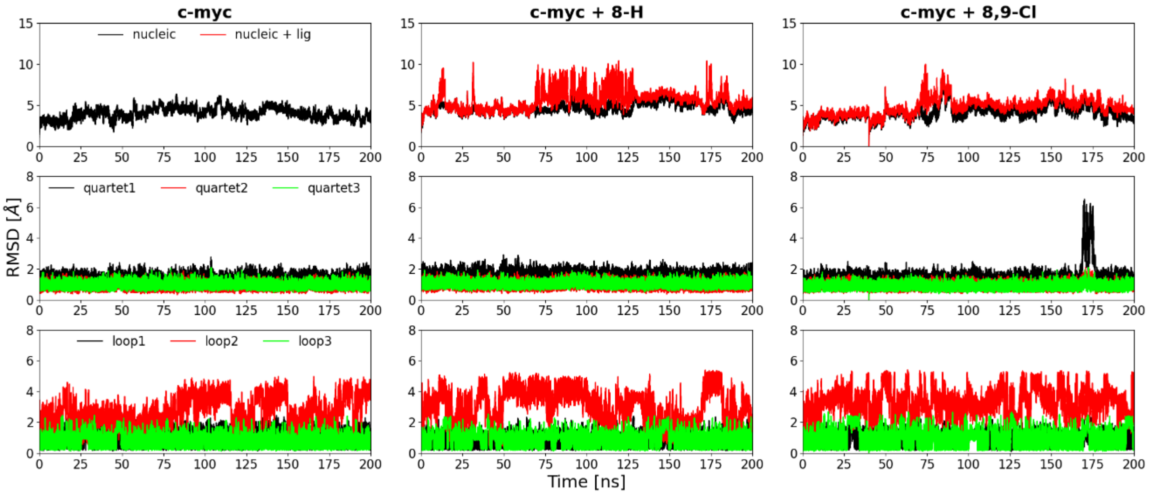

Figure S4. RMSD at 500 K. (sim. 1). Each column refers to a different system: (left column) c-myc without ligand, (central column) c-myc + ligand 8-H and (right column) c-myc + ligand 8,9-di-Cl. Each row represents a different set of heavy atoms selection for the RMSD calculation: First row: in black all DNA residues, in red all residues + ligand. Second row: black, red, and green are the first, the second and the third loop, respectively. Third row: black, red, and green are the first, the second and the third quartet, respectively.

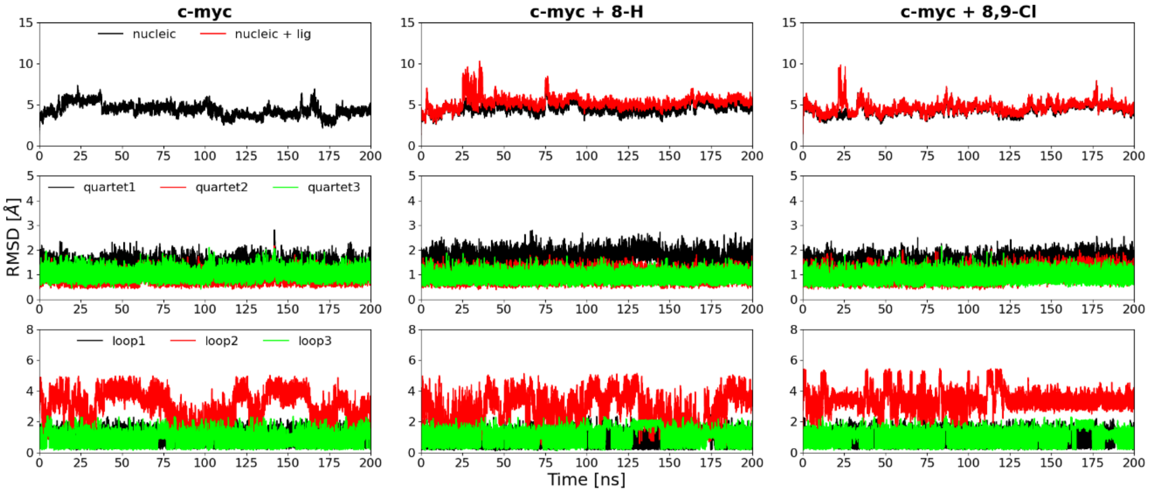

Figure S5. RMSD at 500 K. (sim. 2). Each column refers to a different system: (left column) c-myc without ligand, (central column) c-myc + ligand 8-H and (right column) c-myc + ligand 8,9-di-Cl. Each row represents a different set of heavy atoms selection for the RMSD calculation: First row: in black all DNA residues, in red all residues + ligand. Second row: black, red, and green are the first, the second and the third loop, respectively. Third row: black, red, and green are the first, the second and the third quartet, respectively.

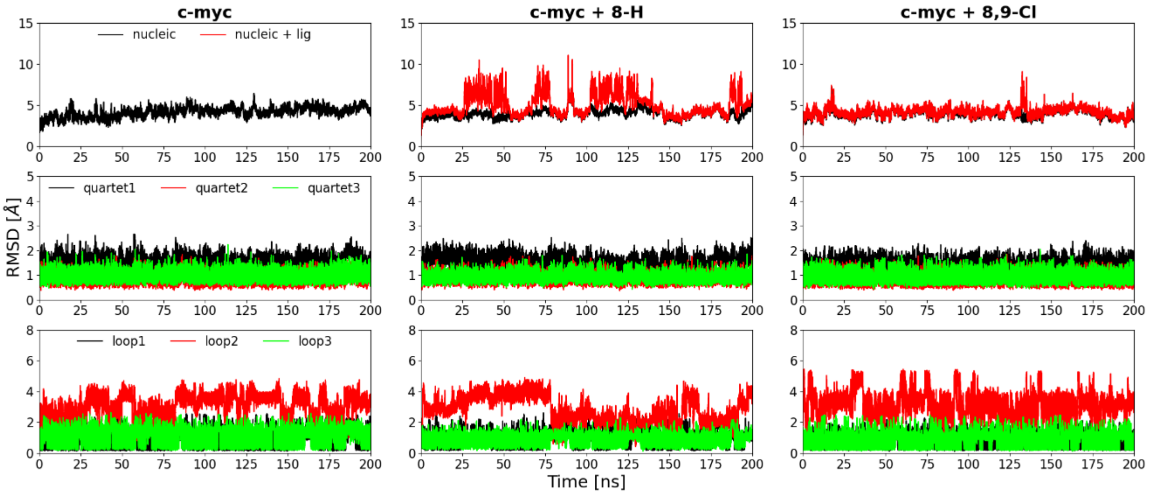

Figure S6. RMSD at 500 K. (sim. 3). Each column refers to a different system: (left column) c-myc without ligand, (central column) c-myc + ligand 8-H and (right column) c-myc + ligand 8,9-di-Cl. Each row represents a different set of heavy atoms selection for the RMSD calculation: First row: in black all DNA residues, in red all residues + ligand. Second row: black, red, and green are the first, the second and the third loop, respectively. Third row: black, red, and green are the first, the second and the third quartet, respectively.

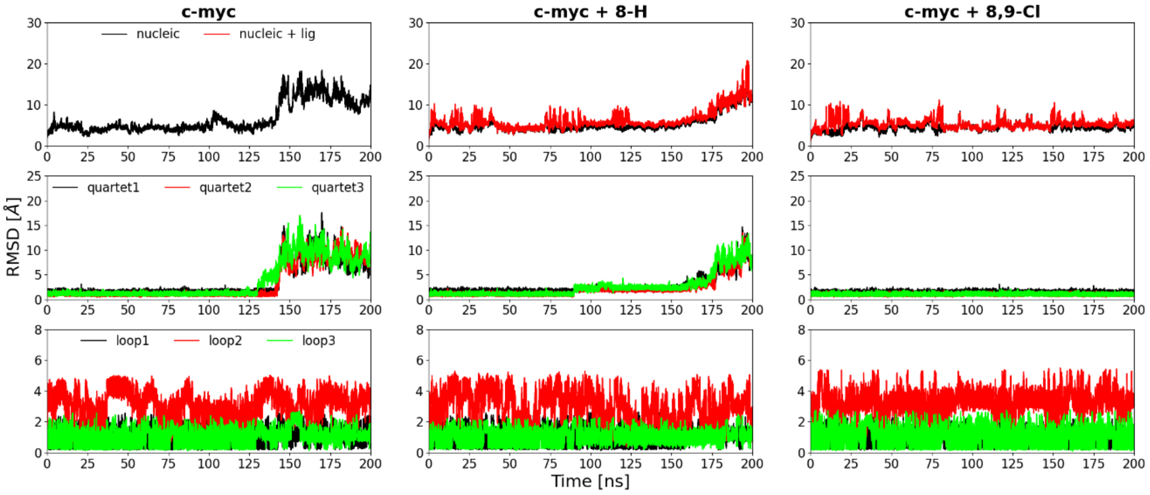

Figure S7. RMSD at 550K. (sim. 1). Each column refers to a different system: (left column) c-myc without ligand, (central column) c-myc + ligand 8-H and (right column) c-myc + ligand 8,9-di-Cl. Each row represents a different set of heavy atoms selection for the RMSD calculation: First row: in black all DNA residues, in red all residues + ligand. Second row: black, red, and green are the first, the second and the third loop, respectively. Third row: black, red, and green are the first, the second and the third quartet, respectively.

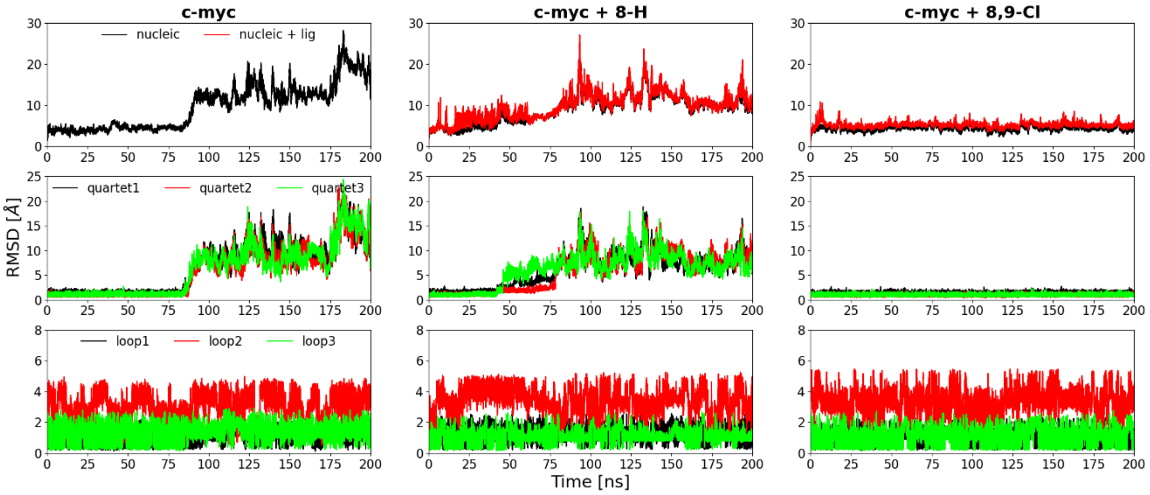

Figure S8. RMSD at 550 K. (sim. 2). Each column refers to a different system: (left column) c-myc without ligand, (central column) c-myc + ligand 8-H and (right column) c-myc + ligand 8,9-di-Cl. Each row represents a different set of heavy atoms selection for the RMSD calculation: First row: in black all DNA residues, in red all residues + ligand. Second row: black, red, and green are the first, the second and the third loop, respectively. Third row: black, red, and green are the first, the second and the third quartet, respectively.

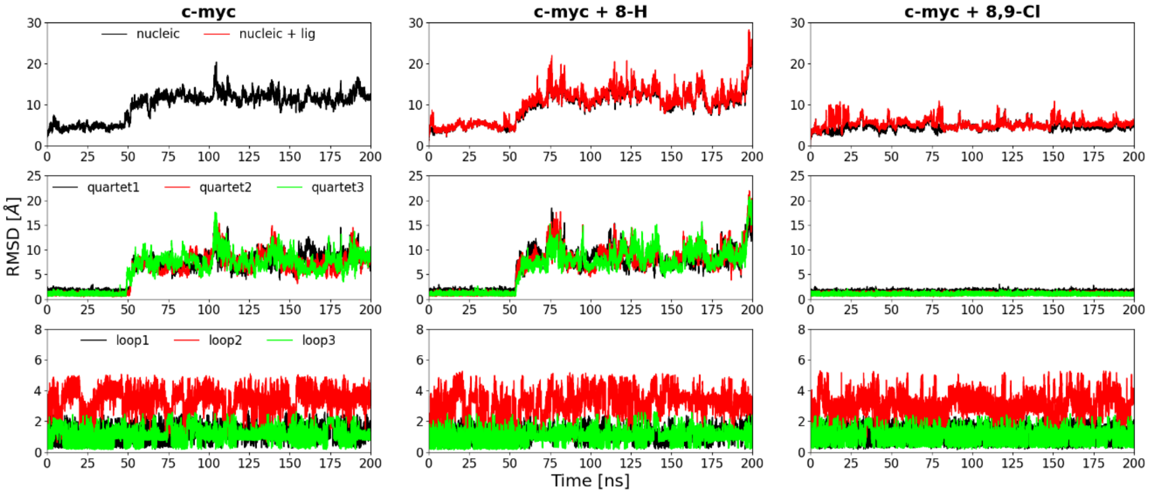

Figure S9. RMSD at 550 K. (sim. 3). Each column refers to a different system: (left column) c-myc without ligand, (central column) c-myc + ligand 8-H and (right column) c-myc + ligand 8,9-di-Cl. Each row represents a different set of heavy atoms selection for the RMSD calculation: First row: in black all DNA residues, in red all residues + ligand. Second row: black, red, and green are the first, the second and the third loop, respectively. Third row: black, red, and green are the first, the second and the third quartet, respectively.

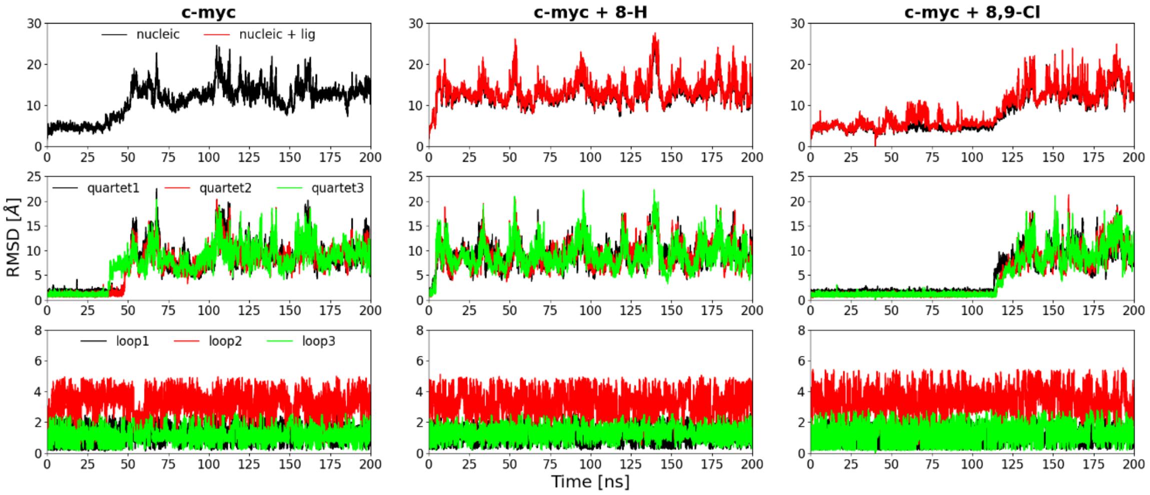

Figure S10. RMSD at 600 K. (sim. 1). Each column refers to a different system: (left column) c-myc without ligand, (central column) c-myc + ligand 8-H and (right column) c-myc + ligand 8,9-di-Cl. Each row represents a different set of heavy atoms selection for the RMSD calculation: First row: in black all DNA residues, in red all residues + ligand. Second row: black, red, and green are the first, the second and the third loop, respectively. Third row: black, red, and green are the first, the second and the third quartet, respectively.

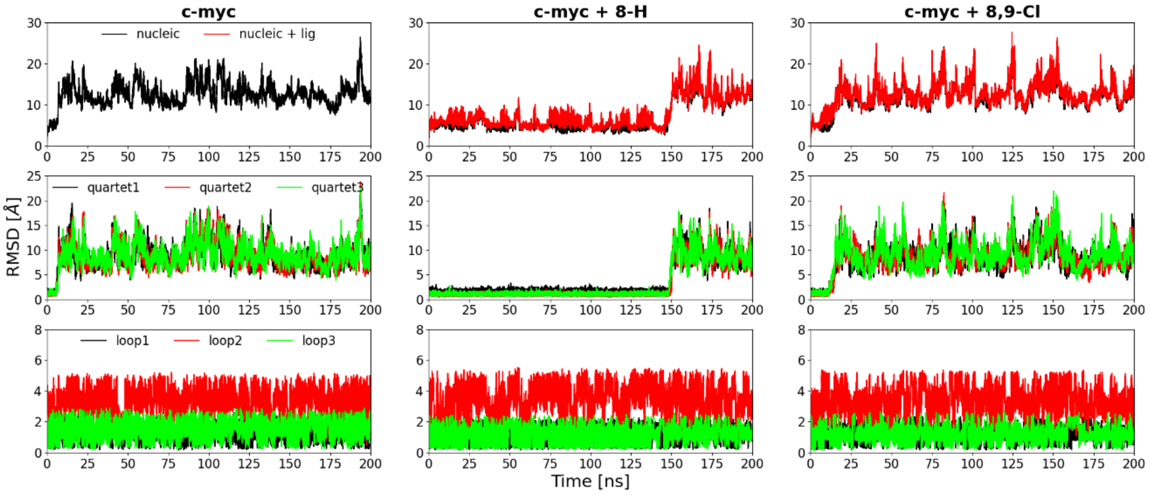

Figure S11. RMSD at 600 K. (sim. 2). Each column refers to a different system: (left column) c-myc without ligand, (central column) c-myc + ligand 8-H and (right column) c-myc + ligand 8,9-di-Cl. Each row represents a different set of heavy atoms selection for the RMSD calculation: First row: in black all DNA residues, in red all residues + ligand. Second row: black, red, and green are the first, the second and the third loop, respectively. Third row: black, red, and green are the first, the second and the third quartet, respectively.

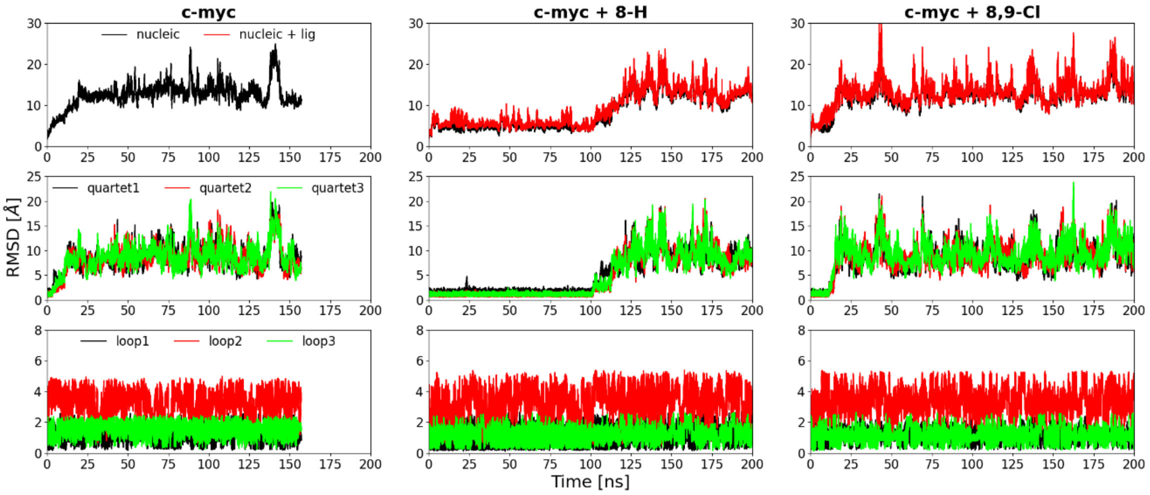

Figure S12. RMSD at 600 K. (sim. 3). Each column refers to a different system: (left column) c-myc without ligand, (central column) c-myc + ligand 8-H and (right column) c-myc + ligand 8,9-di-Cl. Each row represents a different set of heavy atoms selection for the RMSD calculation: First row: in black all DNA residues, in red all residues + ligand. Second row: black, red, and green are the first, the second and the third loop, respectively. Third row: black, red, and green are the first, the second and the third quartet, respectively.

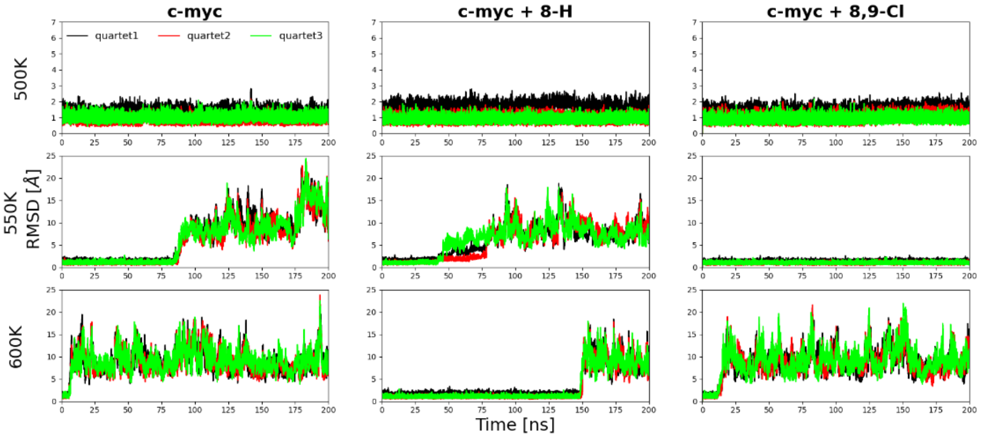

Figure S13. RMSD of the quartets at 500, 550 and 600 K. (sim. 2). Time evolution of the RMSD with respect to the initial structure in the simulation at: 500 K (top row); 550 K (middle row) and 600 K (bottom row), calculated for the quartets of the ligand-free c-myc (left column), the c-myc + 8-H (central column) and c-myc + 8,9-di-Cl complexes.

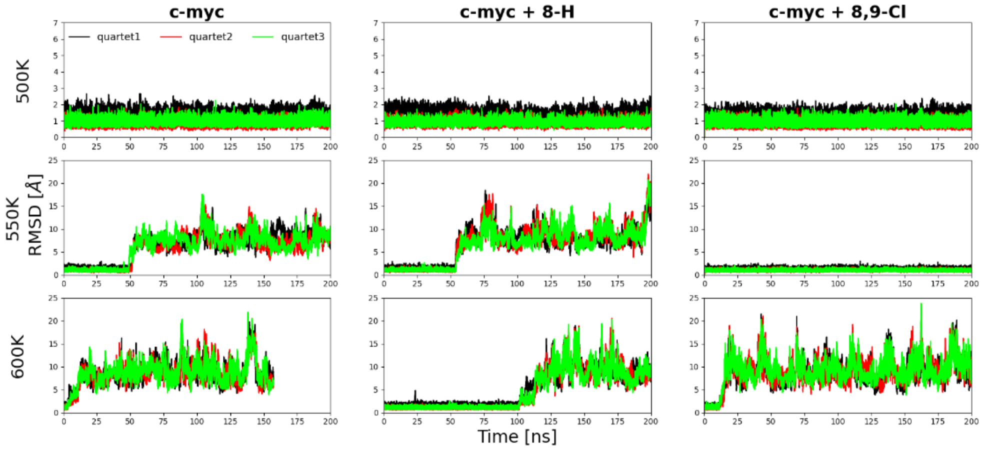

Figure S14. RMSD of the quartets at 500, 550 and 600 K. (sim. 3). Time evolution of the RMSD with respect to the initial structure in the simulation at: 500 K (top row); 550 K (middle row) and 600 K (bottom row), calculated for the quartets of the ligand-free c-myc (left column), the c-myc + 8-H (central column) and c-myc + 8,9-di-Cl complexes.
